# Population Processes at Multiple Spatial Scales Maintain Diversity and Adaptation in the *Linum marginale* - *Melampsora lini* Association

**DOI:** 10.1371/journal.pone.0041366

**Published:** 2012-07-31

**Authors:** Adnane Nemri, Luke G. Barrett, Anna-Liisa Laine, Jeremy J. Burdon, Peter H. Thrall

**Affiliations:** 1 CSIRO Plant Industry, Canberra, Australian Capital Territory, Australia; 2 Metapopulation Research Group, Department of Biosciences, University of Helsinki, Helsinki, Finland; University of Liverpool, United Kingdom

## Abstract

Host-pathogen coevolution is a major driver of species diversity, with an essential role in the generation and maintenance of genetic variation in host resistance and pathogen infectivity. Little is known about how resistance and infectivity are structured across multiple geographic scales and what eco-evolutionary processes drive these patterns. Across southern Australia, the wild flax *Linum marginale* is frequently attacked by its rust fungus *Melampsora lini*. Here, we compare the genetic and phenotypic structure of resistance and infectivity among population pairs from two regions where environmental differences associate with specific life histories and mating systems. We find that both host and pathogen populations are genetically distinct between these regions. The region with outcrossing hosts and pathogens that go through asexual cycles followed by sexual reproduction showed greater diversity of resistance and infectivity phenotypes, higher levels of resistance and less clumped within-population spatial distribution of resistance. However, in the region where asexual pathogens infect selfing hosts, pathogens were more infective and better adapted to sympatric hosts. Our findings largely agree with expectations based on the distinctly different host mating systems in the two regions, with a likely advantage for hosts undergoing recombination. For the pathogen in this system, sexual reproduction may primarily be a survival mechanism in the region where it is observed. While it appears to potentially have adverse effects on local adaptation in the short term, it may be necessary for longer-term coevolution with outcrossing hosts.

## Introduction

Spatial and temporal heterogeneity in the genetic structure of interacting host and pathogen populations is a characteristic of natural host-pathogen systems [1], [2], [3], [4]. It is widely assumed that variation in host resistance and pathogen infectivity plays a crucial role in the spread and persistence of infectious diseases. While spatial variation in resistance is generally observed at all spatial scales studied (reviewed in [5]), it is unclear how this structure integrates hierarchically across the multiple spatial scales of a host-pathogen interaction, eg. within and among host populations of a region and among regions. Similarly, it is still mostly unclear how the spatial structure of pathogen infectivity relates to the spatial structure of host resistance for a given host-pathogen association. As a result, the consequences of spatial variation in resistance and infectivity for disease dynamics and coevolution within and across spatial scales are largely unresolved. In addition, little is known about the processes that contribute to generating this spatial structure, with ongoing debate on the respective roles of coevolution, adaptation to abiotic environments, demographic processes and stochasticity [6], [7], [8]. Hence, contrasting the patterns of spatial variation in resistance or infectivity across a range of spatial scales, and identifying biological or environmental processes that are predicted to generate these patterns constitute major goals in coevolutionary biology.

In nature, individual host-pathogen associations often occur across a range of environments. Patterns of resistance and infectivity within such interactions likely result from the interactive effects of local, population and regional scale processes [5]. Within local host populations, the spatial distribution of individuals and resistance genes may have important consequences for disease epidemiology and resultant patterns of host-pathogen co-adaptation [5], [9]. Specifically, theoretical work predicts that genetically diverse host populations are better at preventing disease spread than more homogeneous ones [10]. This prediction is supported by studies in several natural plant and animal host-pathogen systems [5], [11], [12], [13], [14], [15]. In agricultural systems, experimental comparisons of random crop varietal mixtures and patterned stands infected with various rusts and mildews have demonstrated that host diversity can restrict epidemic development and reduce the selection pressure imposed by pathogens [16], [17], [18]. This benefit of host diversity in limiting disease spread is reduced when pathogen populations exhibit correlated variation in infectivity [19]. Across a metapopulation, differentiation in resistance phenotypes among host populations is also likely to negatively affect the spread of epidemics [20].

A number of processes have been proposed as contributing to the generation of spatial structure in resistance and infectivity, including coevolution and mode of reproduction [9], [21], [22], [23]. At very fine scales, mating system in plants has a major influence on the spatial distribution of traits, with host outcrossing found to generate relatively random spatial distributions among individuals whereas self-fertilization results in more clumped distributions [24], [25]. Assuming no selection by the pathogen acting to disrupt this distribution, we predict that this should also be true for resistance genes. Likewise, on the pathogen side, variation in infectivity can be increased and maintained through sexual recombination [26], [27]. Consistent with this, studies of agricultural rust pathogens also describe sexually reproducing populations to be more diverse than their asexual counterparts (e.g. [28], [29]).

At broader scales, differentiation among populations or regions of host and pathogen should reflect the combined effects of selection, gene flow and drift [30]. Low within-population or region connectivity and adaptive processes resulting from strong local coevolution are expected to increase variation in resistance and infectivity across a region [31]. Furthermore, non-adaptive processes such as random drift, bottlenecks and founder effects should reinforce any differentiation [32]. Additionally, correlated traits evolving as regional adaptations not directly related to antagonistic coevolution may indirectly affect the spatial structure of resistance and infectivity if they affect genetic recombination [33]. Estimating the relative importance and effect of these multiple factors at each spatial scale is crucial to being able to predict the structure of resistance and infectivity in a region and the resulting local coevolutionary trajectories and disease dynamics [6], [34], [35]. However, directly identifying causal factors underlying variation within and among host and pathogen demes has proved difficult as populations often exist among regions where environmental grain, life-history differences and variation in other factors is confounded. A more immediate goal can be to, first, assess the extent of this variation across heterogeneous environments and, second, compare the observed patterns with those predicted by the specific key life-history traits of host and pathogen found in those environments.

In this study we examine patterns of resistance and infectivity at multiple spatial scales in the interaction between the Australian wild flax species, *Linum marginale*, and its host-specific rust pathogen, *Melampsora lini*. At the regional level, evidence has accumulated indicating marked geographic differences in modes of reproduction of both host and pathogen [36], [37]. Allozyme studies revealed that host populations in subalpine (“Mountains”) regions of south-east Australia show a high level of selfing, while those in the adjacent drier plains regions (“Plains”) undergo relatively high levels of outcrossing [36]. However, information regarding the genetic structure of host populations within individual demes is currently missing. The development of *L. marginale* microsatellite markers provides an opportunity to investigate polymorphism in host populations at multiple scales, in the context of regional differences in outcrossing rate. For the pathogen, *M. lini*, an analogous geographic pattern is found in that two genetically and spatially distinct lineages exist. In the Plains region, the dominant lineage shows a mixed mating system (both sexual and asexual reproduction), while that in the Mountains region reproduces almost exclusively asexually [38], [39]. Therefore, the *Linum-Melampsora* interaction in the Plains and Mountains regions provides us a opportunity to explicitly compare the spatial structure of resistance and infectivity at multiple scales across different environments. Specifically, we: (1) estimate regional genetic differentiation in host and pathogen and use reciprocal inoculation trials to test the prediction that the geographical distribution of pathogen lineages cannot be explained by regional adaptation in infectivity alone; (2) verify the prediction that host outcrossing and pathogen sexual reproduction associate with greater phenotypic diversity of host and pathogen within and among regions; and (3) test the prediction that selfing associates with a more clumped fine-scale spatial distribution of resistance than outcrossing but that outcrossing allows the concentration of multiple resistances within some rare individuals.

## Materials and Methods

No specific permits were required for the field studies reported here. Verbal agreement was obtained from Steve Cathcart of the NSW National Parks & Wildlife Services to sample in the Mountains populations. Sampling in Plains populations did not require specific permissions as the sampling was conducted along public roads in locations not privately owned or protected in any way. The field studies did not involve endangered or protected species.

### *Linum marginale – Melampsora lini* Pathosystem

The Australian native flax, *Linum marginale,* is found in a broad range of habitats across southern Australia [40]. Populations are spatially discrete, separated by distances ranging from a few hundreds of metres to tens of kilometres and vary substantially in size from less than 100 to more than 5000 plants [41], while densities can be as high as 40 m^−2^ [42]. To our knowledge, pollination mechanisms are unknown for *L. marginale*, although circumstantial evidence from crosses and introgressed lines suggests the absence of within- or among-population mating incompatibility or self-incompatibility [43].

Infection by the rust pathogen *Melampsora lini* has the potential to cause significant fitness impacts on *L. marginale*, including reduced seed yield and increased host mortality [44]. The rust occurs across the geographic and habitat range of its host. Dikaryotic urediospores constitute the asexual stage of the rust; they are wind-dispersed and their rapid propagation can lead to local epidemics, with up to 8 reproduction cycles in a growing season. This asexual stage is observed in both the Mountains and Plains regions. However, in the Plains but not in the Mountains, it is followed by the sexual stage at the end of the disease season. Infectivity in *M. lini* is controlled by effector genes (also called avirulence genes) which determine successful infection on a given host [45]. Both full and partial resistance to *M. lini* in *L. marginale* result from highly specific gene-for-gene interactions [43]. Partial infections are typically associated with a reduction in the impact of disease on host fitness. In this system, a high frequency of partial resistance indicates lower pathogen local adaptation [46].

**Figure 1:**
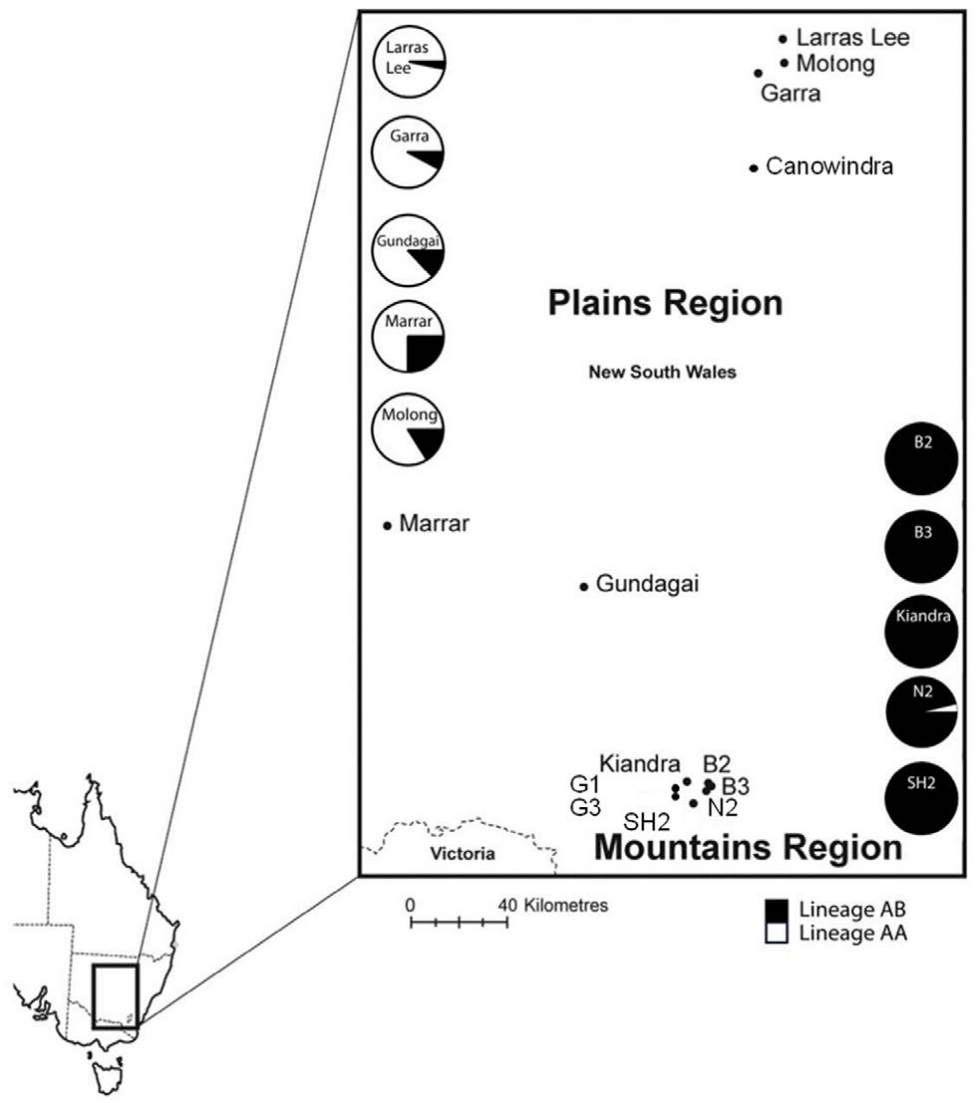
Geographical distribution of *M. lini* lineages in the Mountains and Plains regions in southeastern New South Wales, Australia. Within each region, 5 host-pathogen population pairs were sampled for regional assessments of host resistance and pathogen infectivity. Genetic characterization of pathogen isolates (collected during the 2006 growing season) was conducted using microsatellite markers. On average, 30 pathogen isolates were tested per population.

Genetic analysis of *M. lini* isolates collected from *L. marginale* populations across Australia has revealed the existence of two widespread pathogen lineages (termed AA and AB). These lineages have largely discrete infectivity profiles, separate (although partly overlapping) geographic distributions (Fig. 1) and differ in mode of reproduction [39]. Lineage AB appears to have originated from hybridization between lineage AA and an extinct or as yet unidentified lineage BB [39] and is largely restricted to the Mountains. Consistent with an exclusively clonal mode of reproduction, lineage AB isolates show a fixed pattern of heterozygosity (single A and B alleles at tested microsatellite loci), low genotypic diversity, and strong linkages within and between genetic and phenotypic markers [37]. In contrast, lineage AA strains occur in the drier Plains and have a population structure consistent with mixed sexual and clonal reproduction.

Environmental variation over the geographic range of *L. marginale* plays a critical role in determining the distribution of the two *M. lini* lineages. In the Mountains populations the main host and pathogen growing season corresponds with late spring and summer [36]. Warm to hot temperatures occurring during this season likely restrict the invasion of lineage AA strains into Mountain host populations as such conditions trigger formation of dormant teliospores in this lineage but not lineage AB [39]. Autumn frosts cause plants to die back to protected shoots and rootstocks which generates abrupt crashes in pathogen numbers. The pathogen survives as clonal propagules on plant shoots between growing seasons. In contrast, in the Plains environment (hot, dry summers; mild winters), the main host and pathogen growing season corresponds with the cool winter and spring months. In years where there is a strong summer drought, hosts are annual, or survive hot dry summers as underground rootstocks where above-ground shoots die back. Under such conditions, initiation of the pathogen sexual cycle allows survival as dormant teliospores, favoring the persistence of lineage AA isolates [37], [39]. Isolates from the Mountains and Plains undergo a similar number of clonal cycles of reproduction and increase in a given growing season.

### Host and Pathogen Genetic and Phenotypic Structure within and Among Regions

#### Host and pathogen sampling and genetic characterization

For hosts used for the assessment of resistance diversity in the Mountains vs. Plains regions, seed was collected during the 2005 growing season from 29–34 *L. marginale* individuals from each of 5 Mountains (N2, Kiandra, B2, B3, SH2) and 5 Plains populations (Gundagai, Garra, Larras Lee, Marrar, Molong) (Fig. 1). At the same time, in each of these populations, an average of 30 *M. lini* isolates were sampled haphazardly, each from a single pustule on a unique infected plant, and propagated as described previously [41]. Genetic characterization of these isolates was reported in a previous study [37]. Genetic evidence indicates no ploidy differences between lineages of *M. lini*.

Host populations were genetically characterized using ten microsatellite markers derived from a set developed to study the cultivated flax *Linum usitatissimum* including *Lub*4, *Lub*11, *Lua*58, *Lua*64, *Lua*83B, *Lua*105, *Lua*133, *Lu*139, *Lu*158 and Lu176 (Supplemental Information (SI) Table S1) [47]. PCR amplification consisted of an initial 4 minutes of denaturation at 95°C followed by 15 cycles comprising 30 s of denaturation at 94°C, 30 s of annealing at 65°C and 80 s of elongation at 72°C. A second amplification phase was performed with 15 s of denaturation at 94°C, 15s of annealing at 50°C annealing and 45 s of elongation at 72°C repeated over 30 cycles. The size of the amplicons was determined using an ABI 3130×l sequencer. Fragments were analysed using the Genemapper v4.0 software (Applied Biosystems, Carlsbad, California, USA). Multiple bands were observed at most microsatellite markers tested (Table S1). Hence, we scored the presence/absence of individual bands for each microsatellite marker in binary fashion to produce AFLP-like data for each population (SI Table S2). We used GenAlex [48] to perform an analysis of molecular variance (AMOVA) on our microsatellite data and to estimate Nei’s genetic distance between populations.

#### Host resistance/pathogen infectivity census among and within regions

We assessed phenotypic variation in host resistance within and among regions from the 5 Mountain and 5 Plains populations described above using a set of 12 phenotypically distinct pathogen isolates (Mountains/lineage AB: SH2-1, N2-9, K8, SH2-8, B3-16, B3-4; Plains/lineage AA: Gar-26, Gun-14, Gun-37, L.Lee-49, Mar-34, Mar-41). These pathogen isolates were selected as they showed unique infectivity profiles when inoculated onto a set of 13 differential *L. marginale* lines (see below). Inoculations were performed as previously described [41], [42]. A single plant was established from seed of each host sampled in the field; stems taken from each individual were assessed for their infection-type response to the 12 pathogen isolates. All plant resistance responses were assessed by assigning plants to one of 3 responses to pathogen challenge: 1) resistant, 2) partially resistant, or 3) fully susceptible (Fig. S1). Using this partition into 3 categories, we estimated differences in hosts from the two regions when challenged with sympatric vs. allopatric pathogens. Next, we were interested in comparing similarities in resistance specificities across populations from a region. Hence, we estimated diversity in resistance responses by grouping fully susceptible and partially resistant responses (categories 2 and 3 described above) into a single “infected” category, to provide a contrast with the fully resistant ( =  no sporulation) category. In this analysis, we grouped under a common “resistance phenotype” the individuals that resist the same isolates and get infected by the same isolates. This served to specifically quantify individuals within and among populations that are functionally similar during epidemics, despite slight variations in the degree of resistance.

Similarly, we determined pathogen “infectivity phenotypes” by inoculating the isolates from each host population onto a standard set of 13 *L. marginale* differential lines [43]. These differential lines represent a continental sample of hosts that serve to maximise the diversity of resistance in the set and are hence used to discriminate pathotypes with higher resolving power than hosts sampled from sympatric populations. As we were interested only in diversity, binary infection scores were used. Isolates that were infective on the same hosts and non-infective on the same hosts were grouped within a single “infectivity phenotype”. The number of host differential lines that could be overcome over the total number of hosts assayed gave the average infectivity of an infectivity phenotype.

#### Statistical analyses

Using phenotypic data for the five Mountain and five Plains populations scored against 12 isolates of the two pathogen lineages, we implemented a hierarchically nested Generalized Linear Mixed Model (GLMM) to analyse how host responses to inoculation were distributed across populations and regions, and to detect possible host region x pathogen lineage interactions indicative of local adaptation. The explanatory fixed variables were region (Mountains vs. Plains), host population nested under region, and pathogen lineage (AA vs. AB). Host line (nested under host population × region) and pathogen isolate (nested under lineage) were defined as random variables. Whether the variance associated with a given random effect significantly exceeded zero was evaluated using Wald Z-tests [49]. Non-significant interactions were dropped from models in a step-wise manner. The model was fitted using PROC GLIMMIX in SAS 9.1.3 (SAS Institute 2001). We carried out post hoc pair-wise comparisons to identify which host region x pathogen lineage combinations differed significantly. We used G-tests to determine whether the number of responses in the three categories – susceptible, partially resistant and resistant – differed among host regions and pathogen lineages. We used chi-square tests to assess whether the number of resistance and infectivity phenotypes per region (see definition above) differed between Mountains and the Plains regions, and G-tests to assess whether the number of phenotypes differed among populations within regions.

### Fine-scale Within-population Assessment of Host Genetic and Phenotypic Diversity and Outcrossing Rate

#### Spatial genetic autocorrelations of hosts within transects

For hosts used in the fine-scale estimation of phenotypic and genetic diversity, single two-metre wide transects running through the centre of each of two Mountains (G3, G1) and two Plains (Gundagai, Canowindra) populations were established at the beginning of the 2000 growing season (Fig. 1). As seed capsules matured, the spatial location of every plant in each transect was recorded and seed collected from each plant separately, independent of its infection status. Mountain transects were both 120 m long and sampled 292 and 352 individuals respectively; Plains transects were 80 m long and sampled 352 and 316 individuals respectively. For the four population transects, aggregation of spatial distribution of plants was calculated using Lloyd’s index of patchiness [50], as P = 1+[(*σ* ^2^-*m*)/*m* ^2^] where *m* and *σ* ^2^ are the mean density of the target population in 2 m^2^ blocks and its variance respectively. An index value of less than 1 indicates a random distribution while a value of more than 1 indicates aggregation. This measure is insensitive to overall population density. Genotyping was conducted following the procedure described above for the hosts from the 5 Mountains and 5 Plains populations. The converted AFLP-like genotype data for the four transects was used in the SGS software (v1.0d) [51] to calculate genetic dissimilarity between individuals using Tanimoto distance [52]. Distograms were generated for blocks of 0.5 m for the first 10 metres (1000 permutations using Monte Carlo permutation procedure). The overall mean of genetic distances between all individuals served as reference for random spatial structure and to determine 99% confidence intervals. This process calculates, for each distance block, whether individuals are more similar to each other than expected at random based on mean genetic distance in the whole population. Hence, this measure is insensitive to population density or patchiness of individuals. Due to sampling being done within transects, the diversity sampled in this study was not used to extract information on population demography such as effective population size.

#### Spatial phenotypic autocorrelations of hosts within transects

Individual seedlings were germinated and grown from seed collected from all plants mapped in the four transects. These seedlings were grown in the glasshouse to adult size and then assessed for their infection type response to two Mountains and two Plains pathogen isolates collected from the same or closely adjacent pathogen populations (Mountains isolates: A54, W10; Plains: GR3, Can2). From each actively growing plant, 4 stems of similar age were cut and inoculated with spores from a single isolate following a standard procedure [42]. Infection types for each plant line x pathogen isolate combination were assessed as resistant, partially resistant or fully susceptible. The spatial structure of phenotypes was assessed by calculating a city-block distance [52] using the resistance phenotype to all four isolates as independent quantitative traits (susceptible, partially resistant and resistant were coded 1, 2 and 3 respectively). The mean phenotypic distance between all pairs of individuals belonging to a given distance class was compared to the mean phenotypic distance between all individuals in the population. This measure is also insensitive to overall population density and patchiness of individuals. Using the SGS software (v1.0d) [51], distograms were generated for blocks of 0.5 m for the first 8 metres (1000 permutations using Monte Carlo permutation procedure) and 99% confidence intervals were determined.

#### Estimation of outcrossing rates within transects

To estimate population equilibrium outcrossing rates for the population transects from G1, G3, Canowindra and Gundagai, we selected 5 microsatellite loci (*Lub*4, *Lub*11, *Lua*58, *Lua*64 and *Lua*83B) that raised a pair of alleles that could be scored as codominant in those populations. At each selected locus, for the 2 selected alleles, at least one allele was always present, and in some individuals both alleles were present. For these selected loci and pairs of alleles, the relative frequencies of rare and common alleles were relatively stable across Mountains or Plains populations with approximately 20%–80% ratio. Outcrossing values obtained for each locus were averaged across loci to generate an estimate of population equilibrium outcrossing rate. The outcrossing rate t was derived as t  =  (1–F)/(1+F), where F is the fixation index calculated as F = 1- (*Het*×*N*)/(2×*A_1_*×*A_2_*). In a population at inbreeding equilibrium of *N* individuals genotyped using a selectively neutral microsatellite marker with two alleles *a_1_* and *a_2_*, *Het* is the number of heterozygotes, and *A_1_* and *A_2_* is the number of *a_1_* and *a_2_* alleles in the population respectively.

## Results

### Genetic and Life History Differences among *M. lini* Lineages Associate with Geographic Distribution at Regional Scale and Regional Adaptation of Pathogens to their Hosts

#### Regional genetic differentiation among host and pathogen populations

**Figure 2:**
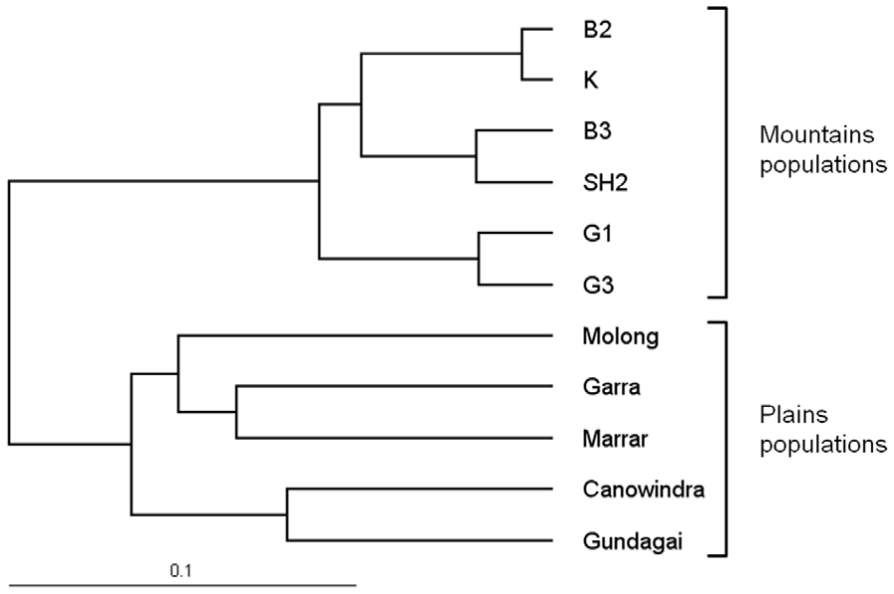
Tree of Nei’s genetic distances within and among *Linum marginale* populations from Mountains and Plains regions.

There was strong genetic differentiation between pathogen lineages in the Mountains and Plains regions respectively. Mountains pathogen populations consisted almost exclusively of lineage AB isolates whereas Plains pathogen populations contained ∼85% lineage AA and ∼15% lineage AB (Fig. 1). Similarly, host populations from Mountains and Plains exhibited strong regional differentiation. Using AMOVA, we found strong structure in the genetic make-up of *L. marginale* populations (*P*<0.01 for all variance components), with most molecular variation contained within individual populations (49%), whereas 34% of the variation was among populations within a region, and the remaining 17% of the variation was explained by the regional origin of the hosts. Estimation of Nei’s genetic distance between host populations showed that populations from the Mountains are genetically closer to each other than to populations from the Plains (Fig. 2). Geographically, Plains populations are separated by much greater distances (up to ∼250 km) than Mountains populations (20 km or less) (Fig. 1). Consistent with this, we found that genetic distances between Plains populations were greater than those between Mountains populations (Fig. 2).

#### Regional adaptation of pathogens to their hosts

**Figure 3:**
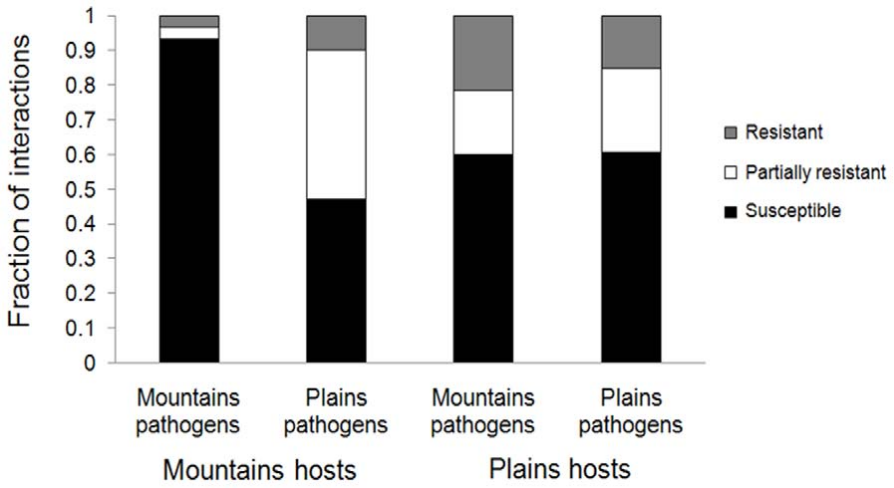
Regional adaptation of Plains and Mountains pathogens to their allopatric and sympatric hosts. Mean patterns of resistance in 5 Mountains and 5 Plains populations of *L. marginale* (30 plants per population) to 6 Mountains and 5 Plains isolates of *M. lini.* Infection type responses shown by hosts were classified into fully resistant, partially resistant or fully susceptible (see Materials & Methods).

Next, we compared the ability of pathogen isolates from Mountains (lineage AB) and Plains (lineage AA) to generate fully resistant, partially resistant or fully susceptible infection type responses on their allopatric and sympatric *L. marginale* plants (Fig. 3; Fig. S2). We found strong evidence for host region × pathogen lineage –interactions driving patterns of resistance (*P*<0.0001; Table 1), suggesting local adaptation of pathogen lineages to their host regions (Fig. 3). Indeed, Mountains isolates showed strong local adaptation as they were found to be more infective on their sympatric hosts than Plains pathogens (local vs. foreign) and more infective on their sympatric hosts than on allopatric hosts (home vs. away; Fig.3; G-test P-values <0.001). Using the home vs. away measure of local adaptation, Plains pathogens appeared locally adapted when comparing their infectivity on Plains hosts vs. Mountain hosts (P<0.0001; Table 1). However, using the local vs. foreign measure of local adaptation, we find that hosts from the Plains are similarly resistant to both pathogen lineages (*P* = 0.729; Fig. 3). This strongly indicates that the low frequency of lineage AB in the Plains is not restricted by its infectivity on the Plains hosts.

**Table 1:** Results of GLMMs on the effects of host origin and pathogen lineage on resistance responses at the regional level.

Variable	df	Residual df	F/P
Host population (withinhost region)	8	305	22.73/<0.0001
Host region	1	305	33.12/<0.0001
Pathogen lineage	1	10	26.31/0.0004
Pathogen lineage × host population	8	3454	30.34/<0.0001
Pathogen lineage × host region	1	3454	244.89/<0.0001

### Within- and among-region Differences in Host and Pathogen Phenotypic Diversity

**Figure 4:**
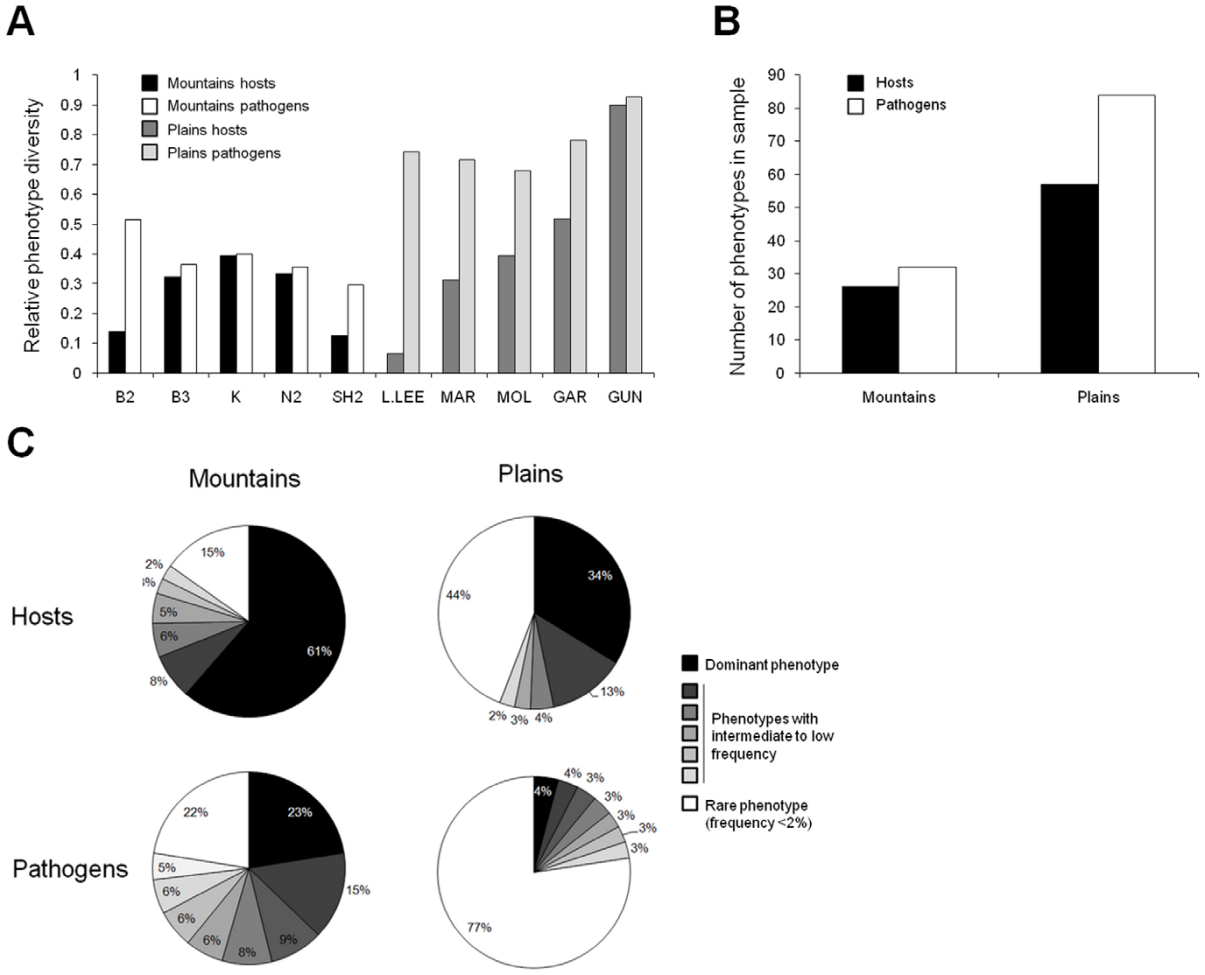
Diversity in resistance of *L. marginale* and infectivity of *M. lini* in Mountains and Plains regions. (A) Relative phenotype diversity was calculated as the ratio of phenotypes found per population over the number of individuals tested. A value of 1 would mean that all individuals tested were different in the assay. (B) The total number of phenotypes in each region calculated for both host and pathogen. (C) The relative frequency in the region of different phenotypes plotted for host and pathogen in Mountains vs Plains. Phenotypes occurring at a frequency of less than 2% were characterized as “rare” and grouped.

For both hosts and pathogens, regional comparisons of resistance and infectivity phenotypes showed significantly greater diversity in Plains than in Mountains populations (chi-square tests, 1 df, Host: *P* = 0.02, Pathogen: *P* = 0.0007). In each region, 5 populations of 30 hosts were assayed, giving a possible maximum of 150 host resistance phenotypes observed (out of theoretically 2^12^ different resistance phenotypes scored binarily using 12 differential pathogen isolates). Out of this possible maximum of 150 resistance phenotypes, 26 were detected in the Mountains and 57 in the Plains of which 11 were common to both. For the pathogen, 32 and 84 pathogen infectivity phenotypes were found in the Mountains and the Plains respectively, of which 8 were common to both (Figs. 4A,B). Surprisingly, at the individual population level, the relative diversity of *L. marginale* populations in the Mountains (average 8.4 resistance phenotypes per population, relative diversity  = 28%) was not significantly different to that of Plains populations (average 13.4 resistance phenotypes per population, relative diversity  = 45%; Student test *P*>0.05; Fig. 4A,B). Since, there is greater regional diversity in the Plains than in the Mountains, this indicates a greater overlap of resistance phenotypes among Mountains host populations, whereas Plains host populations are more differentiated, consistent with the larger geographical distances between these populations compared to Mountains populations. An analysis of phenotypic variance found that host populations within regions did not cluster together (Fig. S3) primarily because there were populations in both regions with little or no resistance (Larras Lee and B2: Fig. S2).

In contrast, individual Mountains pathogen populations were significantly less diverse than their Plains counterparts (Student test, *P*<0.001), being composed of an average of 11.8 infectivity phenotypes while Plains populations contained an average of 22.8 infectivity phenotypes (relative diversity of 39% and 76% respectively; Figs. 4A,B). The Gundagai host-pathogen populations showed the highest diversity with 27 resistance phenotypes out of 30 individuals tested and 25 infectivity phenotypes out of 27 isolates tested. Interestingly, in both Mountains and Plains populations, the number of infectivity phenotypes was always greater than that of resistance phenotypes. Consistent with their lineage origin, an analysis of variance of infectivity found that phenotypically, pathogens from a given region are more similar to each other than to pathogen populations from the other region (Fig. S3).

Within regions, no significant differences were detected in pathogen phenotype diversity levels among populations (G-test, Mountains *P* = 0.85 and Plains *P* = 0.95). However, while no significant differences in resistance phenotype diversity were found among host populations in the Mountains (G-test *P* = 0.34), variation did occur among those in the Plains (G-test, 4 df, *P* = 0.008). One Plains host population in particular (Larras Lee) was almost exclusively composed of a single phenotype that showed complete susceptibility to all isolates tested. Surprisingly, the associated pathogen population diversity was high, with 23 pathotypes out of 31 tested. Similarly, all 30 hosts representing the Garra population could be infected by sympatric isolate Gar-26, yet the Garra pathogen population was very diverse, with 25 infectivity phenotypes detected among the 32 isolates tested. Consistent with these observations, there was no correlation between the number of resistance phenotypes and the number of infectivity phenotypes in either the Plains or the Mountains.

There was a major difference in the occurrence of rare resistance phenotypes (those with frequencies <2%) which represented 15% and 44% of the Mountains and Plains populations respectively (Fig. 4C). Similarly, no one infectivity phenotype comprised more than 4% of the combined Plains region, whereas the most common infectivity phenotype in the Mountains comprised 23% of the isolates from that region (Fig. 4C).

### Regional Differences in Distribution of Resistance Among Individuals of a Population

**Figure 5:**
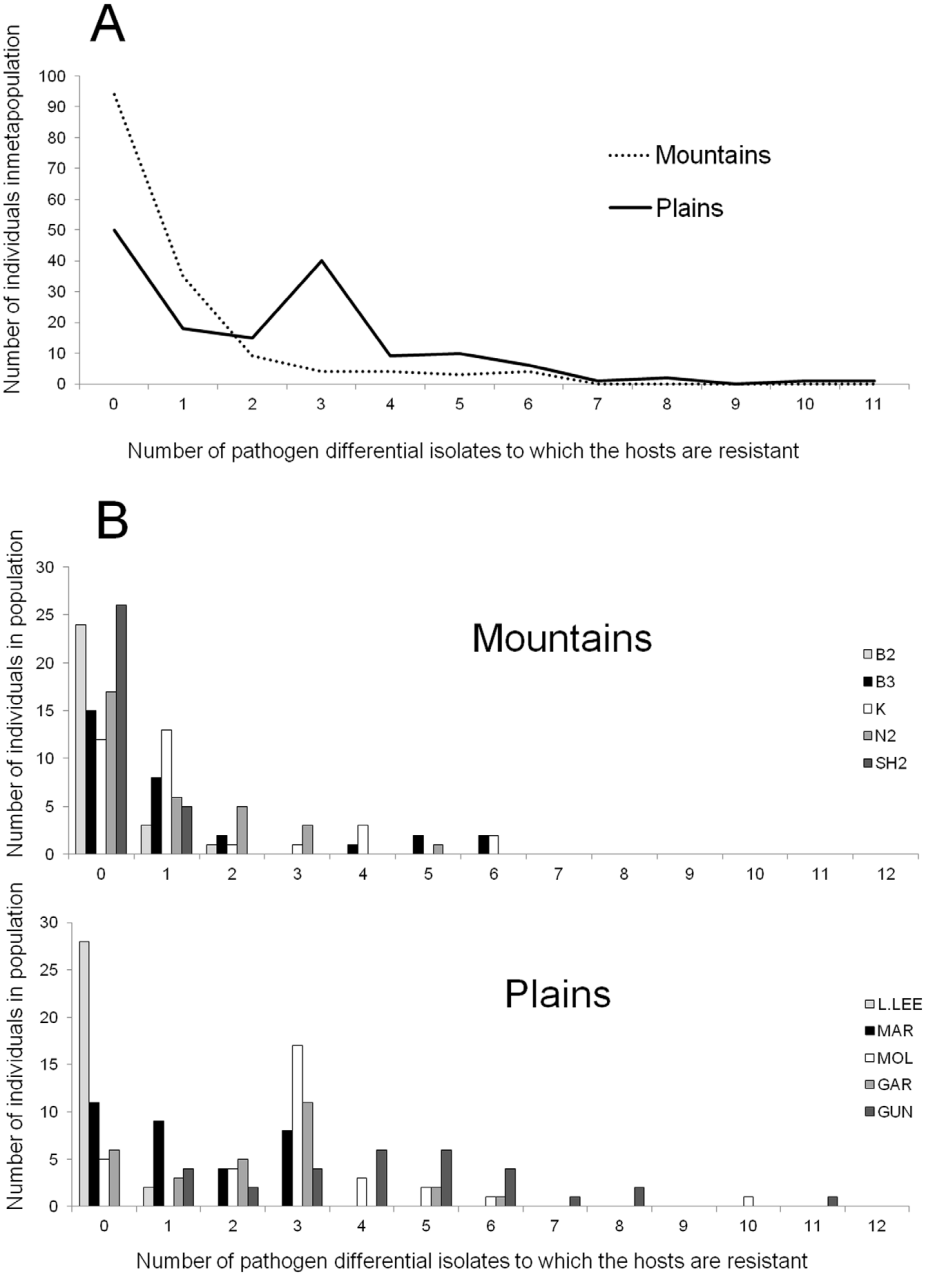
Regional differences in distribution of resistance to *M. lini* in populations of *L. marginale.* An average of 30 individual hosts from each of 5 Mountains and 5 Plains populations were challenged with 12 differential *M. lini* isolates. For each population from Mountains (top panel) and Plains (bottom panel), the number of differential isolates to which individual hosts showed resistance is reported, with 0 meaning the hosts were susceptible to all pathogen isolates and 12 meaning the hosts were resistant to all pathogen isolates tested.

We were further interested in regional differences in the distribution of individual resistance responses within local host populations. We therefore assessed the identity and number of resistant responses observed when plant lines from Mountains and Plains populations were challenged by the set of 12 differential pathogen isolates. There were marked differences among regions in the distribution of number of resistances per individual host. Plains individuals that could resist 3 pathogen differentials were more prevalent than those that could recognise 1 or 2 isolates, but less prevalent than individuals that recognised none of the pathogen isolates (Fig. 5). In contrast, in the Mountains region, most individuals had no resistance to the differential isolates tested and there was a continuous decline in the relative abundance of individuals able to recognize a broader range of isolates (Fig. 5 top panel). However, in the Plains, this negative relationship was less pronounced (and primarily driven by complete susceptibility in the Larras Lee population), and 3 populations out of 5 predominantly contained individuals that could resist 3–4 differential isolates (Molong, Garrar, Gundagai; Fig. 5 bottom panel). In the Plains host populations Molong and Gundagai, some individuals were resistant to as many as 10 and 11 of the 12 differential isolates respectively. In summary, Plains hosts had broader resistance specificities on average than Mountains hosts; in addition, the distribution of resistance across individuals within populations was also different between Plains and Mountains.

### Regional Differences in Patterns of Genetic and Phenotypic Similarity of Hosts at Very Fine Spatial Scales

**Figure 6:**
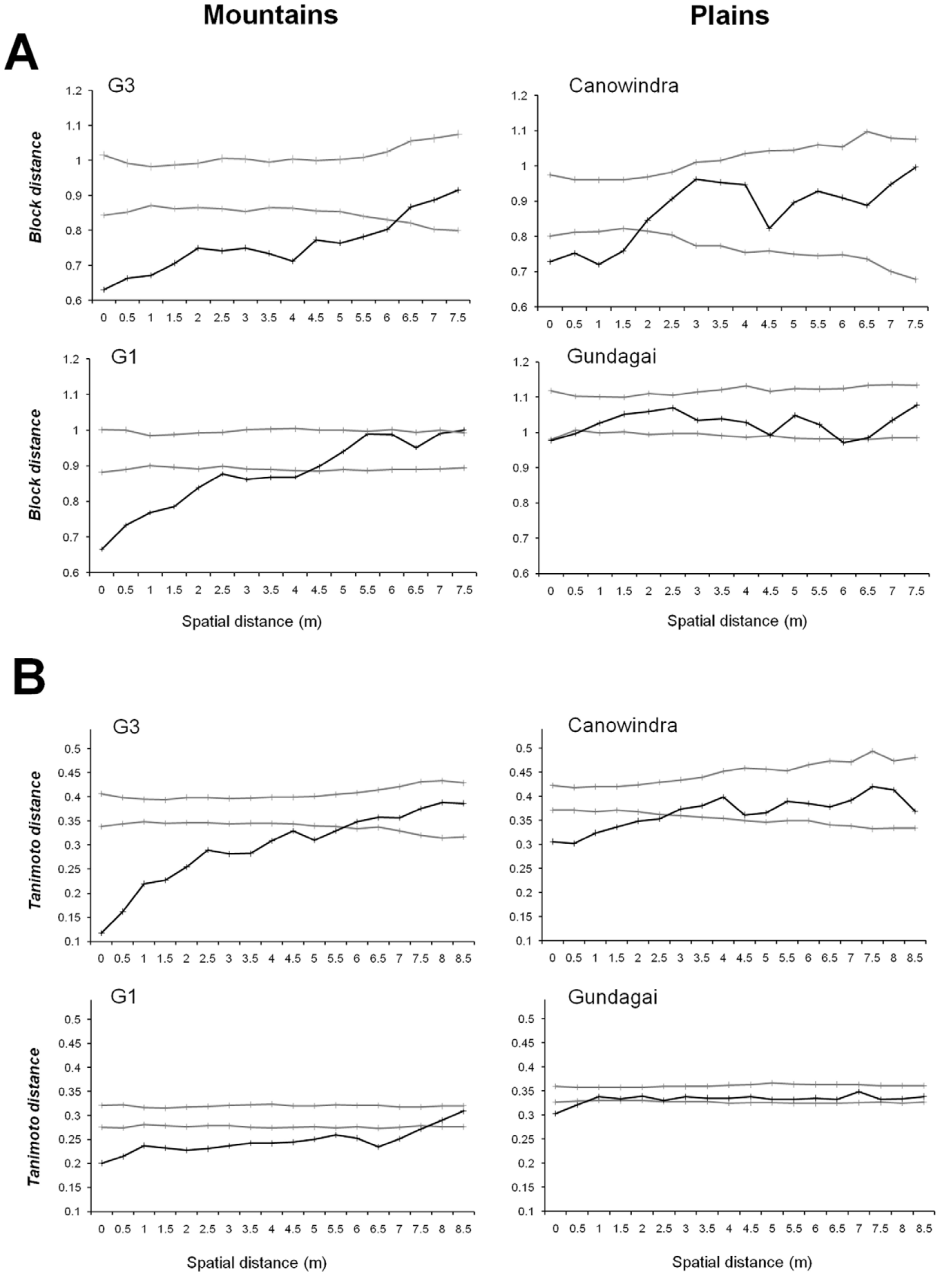
Fine-scale spatial distribution of variation among *L. marginale* individuals in two representative Mountains and Plains populations. (A) Spatial autocorrelations of resistance phenotypes (Block distance) were calculated using infection type responses of individuals to 4 pathogen isolates as quantitative traits. (B) Spatial autocorrelations of genetic structure (Tanimoto distance) were calculated using genotype data from 9 microsatellite markers. Distograms were generated for blocks of 0.5 m for the first 8 and 9 metres for resistance phenotypes (A) and genotypes (B) respectively (1000 permutations). Grey lines represent the upper and lower limit of the 99% confidence intervals, within which a spatial autocorrelation of random datapoints resides. The phenotypic (A) and genotypic distances (B) between individuals in transects are depicted with the black line. Datapoints below the lower limit of the 99% confidence interval indicate the distance at which patches are more similar in resistance (A) or genetically (B) than predicted by random distribution.

Next, we investigated whether differences in the distribution of resistance specificities (Fig. 5) between Mountains and Plains host populations was associated with a difference in the spatial distribution of resistance within populations. We selected two representative populations from the Mountains (G1 and G3) and two from the Plains (Canowindra and Gundagai) to establish transects. The average population density in Plains transects (2.08 plants per m^2^) was higher than in Mountains transects, with 1.33 plants per m^2^ (Student test, *P*<0.05). Individual plants from both Mountains and Plains transects show significant levels of aggregation (Student test, H_0_:µ = 1, *P*<0.01), but no significant differences between populations (Student test, *P*>0.05). Lloyd’s index of patchiness was 2.2 and 2.7 for Mountains populations G1 and G3 respectively and 2.6 and 3.6 in Plains populations Canowindra and Gundagai. The fine-scale distribution of resistance phenotypes showed considerable differences between the two Mountains and Plains transects but not between transects from the same region (Fig. 6A). In the two Mountains populations, individual resistance exhibited a distinctly aggregated distribution with the probability of plants having a similar phenotype (99% confidence) remaining significant up to 6.7 m and 4.7 m in G3 and G1 respectively. In contrast, the Gundagai population showed virtually no spatial structuring, while that at Canowindra was restricted to less than 2 metres (Fig. 6A). In essence, within a patch of plants, groups of individuals with similar resistance phenotypes were considerably larger in the selfing Mountains populations than in the outcrossing Plains populations.

We contrasted this assessment of the spatial distribution of resistance, i.e. a trait potentially under selection, with a parallel evaluation of the spatial distribution of 9 neutral microsatellite markers using the same plants (Fig. 6B). The patterns detected were similar to those based on the distribution of resistance phenotypes. Both Mountains populations showed evidence of significant aggregation of particular microsatellite phenotypes at spatial scales that were very similar to those detected for resistance (both exceeding 5.5 m) while spatial structuring in both the Plains populations was very low (less than 3 and 1 m for Canowindra and Gundagai respectively; Fig. 6B).

Consistent with differences in the spatial distribution of resistance phenotypes and microsatellite genotypes, genetic analysis revealed significant differences in mating system between the transect populations from Mountains and Plains. Mountains populations G1 and G3 demonstrate a high level of inbreeding with outcrossing rates of 1.9% and 2.9% respectively (Fig. S4). In contrast, Plains populations Gundagai and Canowindra exhibit outcrossing rates of 24% and 29% respectively. Previous work using allozymes on this system indicates that these estimates are representative of the other Mountains and Plains host populations included in this study [36].

## Discussion

Identifying the processes that shape the distribution of genetic variation at different spatial and temporal scales represents a key challenge in ecology and evolutionary biology. A general prediction is that coevolution would tend to select host traits that reduce spread of disease and pathogen traits that increase adaptation to sympatric hosts [53]. Also, we predict that non-coevolutionary processes, e.g. adaptation to the physical environment, that affect genetic recombination or gene flow will indirectly affect levels and diversity of host resistance and pathogen infectivity; although the direction of this effect is yet unclear. To examine how diversity is partitioned among different spatial scales, and to attempt to tease apart the processes responsible for structuring genetic variation, we investigated hierarchical patterns of genetic structure as well as patterns of resistance and infectivity in the *Linum-Melampsora* interaction in two geographic regions. These regions represent distinct environments that are also congruent with major differences in life histories of both the host and the pathogen. Here, we attempt to reconcile our findings with patterns predicted as a result of the distinct host mating systems and pathogen modes of reproduction (sexual *vs.* asexual) found in the two environments investigated.

The regional differentiation in pathogen lineages can largely be explained by the different physiologies of these lineages and the different host life histories in these regions. Mountains pathogen populations are almost exclusively composed of lineage AB isolates while Plains populations contain predominantly lineage AA isolates. Lineage AB isolates are found at low frequency (∼15%) in Plains populations but despite similar infectivity to lineage AA isolates likely cannot persist in that environment. Indeed, previous work showed that lineage AB isolates do not readily initiate a sexual cycle and form teliospores in response to warm to hot temperatures [37]. Teliospores, in addition to being vehicles for meiosis, are sclerotic stages that enable *M. lini* in the Plains to survive extended periods of heat and drought during summer after host plants have died back [39], [54]. Conversely, in the Mountains, plants are perennial but grow from late spring through summer. There, telia formation in response to warm to hot temperatures in lineage AA but not lineage AB could have a strong negative effect on both transmission and long-term survival of lineage AA, resulting in a competitive advantage for lineage AB isolates in such environments [37].

If sexual reproduction in the Plains pathogens is primarily a survival mechanism, then the high diversity in infectivity observed there may not be a direct result of coevolution with resistance although it is likely to impact coevolutionary trajectories. Several observations tend to suggest that much of the variation in infectivity observed in some Plains populations may be inconsequential for local disease dynamics. First, we did not find a correlation between the numbers of resistance and infectivity phenotypes within populations. For example, high pathogen diversity was found in Larras Lee despite little or no host resistance, indicating that phenotypic variation may persist in what appears to be a coevolutionary coldspot. Similarly, in Garra, we found a pathogen isolate that could infect every tested host individual from that population and yet that isolate did not dominate the pathogen population. Second, we find that Plains pathogens show weak local adaptation to their hosts, although this result was dependent on the test used. When using the home vs. away metric of local adaptation, i.e. infectivity on sympatric vs. allopatric hosts [55], both pathogen lineages appear locally adapted. However, when using the local vs. foreign metric of local adaptation [56], i.e. infectivity of Plains vs. Mountains pathogens on Plains hosts, local adaptation is detected for Mountains pathogens but not for the Plains pathogens perhaps as a result of the higher resistance of hosts in the Plains. Sexual reproduction in Plains pathogens (lineage AA) provides a mechanistic explanation for their lower degree of local adaptation than that of Mountains pathogens (lineage AB). Recombination in lineage AA isolates may result in the breakdown of co-adapted gene complexes that facilitate regional patterns of adaptation, or dominance effects associated with heterozygosity of *Avr* genes in Plains pathogens (non-infectivity is typically dominant over infectivity) [57]. For example, sexual reproduction between two infective races can generate non infective or partially infective races through breakdown of the infectivity gene complement (incl. effector and inhibitor genes) during meiosis [54].

Hence, sexual reproduction seems required for survival in the Plains and potentially fuels pathogen diversity there despite the lack of resistance of some host populations. When local resistant hosts exist, recombination of infectivity genes may also generate pathogen isolates that are locally unadapted, suggesting an immediate negative effect on coevolution. Yet, this process may be important to maintain infectivity on Plains hosts over longer coevolutionary time. Plains hosts are short-lived and outcrossing whereas in the Mountains they are perennial and inbred. The former life-history combination could result in Plains hosts exhibiting a large and constantly shifting number of resistance gene combinations, as has been suggested in other systems [27]. Higher diversity of resistance genes will drive negative frequency-dependent selection on corresponding pathogen infectivity genes [58], [59]. Previous work has shown that Mountains populations exhibit a significant amount of year-to-year change in their resistance phenotypes [60]. We do not have data the equivalent data from Plains populations, but we predict a greater year-to-year variation of resistance phenotypes than in the Mountains, despite the fact that the number of resistance phenotypes present in any year appears the same in both regions. In this scenario, sexual reproduction in lineage AA would enable the pathogen to both persist in the Plains environment and generate sufficient diversity in infectivity to track Plains hosts on a year-to-year basis. Overall, this indicates an important role for pathogen life-history in driving the spatial distribution of lineages across regions and the patterns of diversity in infectivity found in those regions. Though, we cannot rule out a contribution of non-adaptive processes in structuring diversity of infectivity between pathogen lineages. Consistent with the large geographical distances separating Plains populations, we find that these demes are highly differentiated, suggesting little or no gene flow between them. Random genetic drift may thus at least partly explain higher diversity in lineage AA pathogens while bottlenecks and founder effects may contribute to the lower diversity in lineage AB pathogens in the Mountains where off-season pathogen survival in local host populations appears to be less certain. Generally, our data exemplify that it can be difficult to separate adaptive and non-adaptive processes that may contribute to diversity in infectivity. Hence, future studies that aim to examine the maintenance of host-pathogen diversity in natural systems will likely require specific experimental designs that measure the amount of gene flow between host and pathogen populations, and reciprocal changes in infectivity and resistance across time in host-pathogen population pairs.

With regard to the host, we find clear differences between Mountains and Plains in (1) diversity of resistance and frequency of partial resistance, (2) overall level of resistance; and (3) within-population distribution of resistance. While we cannot discard the existence of regional differences in strength of coevolution or demographic processes, we argue that the consideration of regional differences in mating system, i.e. selfing in the Mountains and outcrossing in the Plains, can reconcile all three results on the host.

First, outcrossing in the Plains should sustain heterozygosity over time whereas selfing in the Mountains tends to produce inbred homozygotes. Partial resistance to flax rust is common in *L. marginale* [46]. Among the many genetic factors that can explain it, incomplete dominance of single resistance genes in heterozygotes [43] or the breakdown of co-adapted loci of additive effect through recombination can explain the observation that partial resistance is more prevalent in outcrossing populations than in their selfing counterparts. Our findings agree with empirical reports that inbreeding populations of *Arabidopsis lyrata* are less resistant and have fewer partial resistance to white blister rust than their outcrossing counterparts [61]. However, they differ from theory predicting that, assuming a single resistance gene shows complete dominance and no associated fitness cost, selfing rate has no effect on the level of resistance in the host population [62]. This discrepancy may be due to the multigene basis of resistance in our experimental system, including some incompletely dominant genes [43].

Second, outcrossing has the potential to result in the aggregation of multiple resistance genes within individual genomes. We find that more rare genotypes concentrating a large number of resistance specificities are found in the Plains environment. This results from either selection of individual genes with broad resistance specificities or the aggregation of several genes each with distinct resistance specificity. The low frequency of those individuals with broad resistance suggests the latter. Theory predicts that an increase in the average number of resistance genes per individual will result in reduced disease prevalence, consistent with observations in the *L. marginale* - flax rust system [20]. Under those circumstances, we predict a selective advantage for those rare host genotypes with high resistance and the maintenance of sexual reproduction (i.e. recombination) in natural host populations to generate them through time, consistent with other empirical and theoretical studies [63], [64], [65], [66]. In addition to reshuffling existing resistance genes, recombination can generate novel resistance alleles, as was shown in cultivated flax where intragenic recombination events in the *L* gene were shown to contribute to the gain and loss of flax rust resistance specificities [67], [68], [69]. In future studies, we aim to survey temporal changes in host resistance diversity to test the hypothesis of a rapid turn-over of resistance phenotypes or that new resistance phenotypes are being generated each year in the Plains populations but not in the Mountains populations.

Third, at the local scale, plants in the Mountains populations tended to be more similar within a patch than plants from the Plains. This pattern held true both for neutral genetic markers and resistance phenotypes. In the Mountains, considering the high rate of inbreeding, we expected strong linkage disequilibrium between resistance genes and microsatellites, consistent with the observed spatial autocorrelations. However, in the Plains, due to outcrossing, we expected links between phenotype and genotype to be less pervasive, contrary to observations. The observed matching patterns of spatial autocorrelation between resistance genes and microsatellites in the Plains suggest that selection is not structuring the spatial distribution of resistance at such fine scales. We found that relatedness of individuals within a patch was significantly greater in the Mountains than the Plains. Assuming equal passive seed dispersal in both regions, the simpler explanation for this observation is differences in pollen movement, i.e. selfing in Mountains vs. outcrossing in Plains. It is unclear how the aggregation of genetically similar plants in a patch influences epidemics in this system, considering *M. lini* spores are thought to be wind dispersed over long distances. To our knowledge, no information is available on how the spatial distribution of resistance alleles affects disease spread within individual populations of natural systems. However, evidence from agricultural systems suggests that epidemics in rusts are negatively affected by cultivar mixtures [17]. A similar effect may be acting in our study populations, as a near-significant (*P*  = 0.08) negative correlation was detected in epidemic years between phenotypic diversity of resistance and disease prevalence in local Mountains populations [20]. Furthermore, a survey of *M. lini* in a Mountains pathogen population has found evidence of local spatial structure whereby the population was dominated by a single major pathogen phenotype spread throughout the host population, while low-frequency phenotypes showed some local clumping [41], [42]. Presumably, this results from the aggregation of genetically similar hosts in the Mountains.

Much of existing coevolutionary theory addresses the evolution and maintenance of host sexual reproduction and mating strategies and predicts an important role for the selective pressure imposed by pathogens [70], [71], [72], [73], [74]. Our data suggest that, by undergoing recombination, the host may also benefit from a pervasive spatial distribution of resistance and the aggregation of multiple resistances in some individuals. This benefit of recombination may also be reinforced by life histories such as annuality rather than perenniality. In the case of the pathogen, strong associations between pathogen specific life histories, including mode of reproduction, and specific environments are also common [75]. Yet, the driving forces favoring clonal vs. sexual reproduction in pathogens are little understood [26]. Our data suggest that sexual reproduction in *M. lini* serves primarily as a survival mechanism but that it may also represent an adaptive response to outcrossing in the host. Taken together, we find that the geographic distributions of those host and pathogen life histories in this natural system potentially have important consequences for the geographic structure of resistance and infectivity at multiple spatial scales. In turn, we postulate that this structure of resistance and infectivity is an essential factor driving disease dynamics and coevolutionary trajectories. Towards being able to deconstruct the often complex selective pressures at work in host-pathogen genetic associations, establishing more such links represents an important goal for coevolutionary biology.

## Supporting Information

Figure S1 **Differential responses to inoculation of wild flax (** ***Linum marginale*** **) genotypes with a flax rust (** ***Melampsora lini*** **) isolate.**
(TIF)

Figure S2 **Regional adaptation of pathogen to their hosts.** *Linum marginale* individual hosts from 5 Mountains (left panel) and 5 Plains populations (right panel) were inoculated with 6 *Melampsora lini* isolates from the Mountains (from left to right in columns: B3-4, B3-16, K8, N2-9, SH2-1, SH2-8) and 6 isolates from the Plains (from left to right in columns: L.Lee-19, Mar-34, Mar-41, Gar-26, Gun-14, Gun-37). Infections were scored as resistant (white squares), partially resistant (grey squares) and fully susceptible (black squares).
(TIF)

Figure S3 **Phenotypic variance and genetic distances in hosts and pathogens populations.** Left panels: Analyses of phenotypic variance in *Linum marginale* populations (top panels) and *Melamposora lini* populations (bottom panels) from Mountains and Plains regions. All components of variance are significant at p<0.01. Right panels: trees of Nei’s phenotypic distances within and among Mountains and Plains populations were generated using UPGMA.
(TIF)

Figure S4 **Estimates of outcrossing rates in two representative Mountains and Plains populations.** Plots show the estimated population equilibrium outcrossing rates for Mountains populations G1 and G3 and Plains populations Gundagai and Canowindra calculated using 3–4 markers and the standard error of the mean.
(TIF)

Table S1 **Nucleotide sequences of microsatellites markers used in this study and size in bp of polymorphic alleles in the** ***L. marginale*** **populations assayed.**
(PDF)

Table S2 **Plains and Mountains** ***L. marginale*** **populations used in this study with indices of genetic variability and polymorphic alleles for each population.**
(PDF)

## References

[1] Thrall PH, Burdon JJ, Young A (2001) Variation in resistance and virulence among demes of a plant host-pathogen metapopulation. Journal of Ecology 89: 736–748.

[2] Burdon JJ, Jarosz AM, Kirby GC (1989) Pattern and patchiness in plant-pathogen interactions- causes and consequences. Annual Review of Ecology and Systematics 20: 119–136.

[3] Laine A-L (2009) Role of coevolution in generating biological diversity: spatially divergent selection trajectories. Journal of Experimental Botany 60: 2957–2970. 19528527 10.1093/jxb/erp168

[4] Salvaudon L, Giraud T, Shykoff JA (2008) Genetic diversity in natural populations: a fundamental component of plant-microbe interactions. Current Opinion in Plant Biology 11: 135–143. 18329329 10.1016/j.pbi.2008.02.002

[5] Laine A-L, Burdon JJ, Dodds PN, Thrall PH (2011) Spatial variation in disease resistance: from molecules to metapopulations. Journal of Ecology 99: 96–112. 21243068 10.1111/j.1365-2745.2010.01738.x PMC3020101

[6] Thompson JN (2005) The geographic mosaic of coevolution. Chicago: University of Chicago Press. 370 p.

[7] Laine A-L, Tellier A (2008) Heterogeneous selection promotes maintenance of polymorphism in host-parasite interactions. Oikos 117: 1281–1288.

[8] Wolinska J, King KC (2009) Environment can alter selection in host-parasite interactions. Trends in Parasitology 25: 236–244. 19356982 10.1016/j.pt.2009.02.004

[9] Barrett LG, Thrall PH, Burdon JJ, Linde CC (2008) Life history determines genetic structure and evolutionary potential of host-parasite interactions. Trends in Ecology & Evolution 23: 678–685. 18947899 10.1016/j.tree.2008.06.017 PMC2653456

[10] Lively CM (2010) The effect of host genetic diversity on disease spread. American Naturalist 175: E149–E152. 10.1086/652430 20388005

[11] Lively CM, Craddock C, Vrijenhoek RC (1990) Red Queen hypothesis supported by parasitism in sexual and clonal fish. Nature 344: 864–866.

[12] Schmid-Hempel P, Crozier RH (1999) Ployandry versus polygyny versus parasites. Philosophical Transactions of the Royal Society of London Series B: Biological Sciences 354: 507–515.

[13] Whiteman NK, Matson KD, Bollmer JL, Parker PG (2006) Disease ecology in the Galapagos Hawk (*Buteo galapagoensis*): host genetic diversity, parasite load and natural antibodies. Proceedings of the Royal Society B: Biological Sciences 273: 797–804. 16618672 10.1098/rspb.2005.3396 PMC1560217

[14] Altermatt F, Ebert D (2008) Genetic diversity of *Daphnia magna* populations enhances resistance to parasites. Ecology Letters 11: 918–928. 18479453 10.1111/j.1461-0248.2008.01203.x

[15] Whitehorn PR, Tinsley MC, Brown MJF, Darvill B, Goulson D (2011) Genetic diversity, parasite prevalence and immunity in wild bumblebees. Proceedings of the Royal Society B: Biological Sciences 278: 1195–1202. 20926436 10.1098/rspb.2010.1550 PMC3049068

[16] Chin KM, Wolfe MS (1984) The spread of *Erisyphe graminis* f. sp. *hordei* in mixtures of barley varieties. *Plant Pathology* 33: 89–100.

[17] Mundt CC (2002) Use of multiline cultivars and cultivar mixtures for disease management. Annual Review of Phytopathology 40: 381–410. 10.1146/annurev.phyto.40.011402.113723 12147765

[18] Mundt C, Brunet J, Sackett K (2008) Impact of density and disease on frequency-dependent selection and genetic polymorphism: experiments with stripe rust and wheat. Evolutionary Ecology 22: 637–657.

[19] Ganz HH, Ebert D (2010) Benefits of host genetic diversity for resistance to infection depend on parasite diversity. Ecology 91: 1263–1268. 20503859 10.1890/09-1243.1 PMC3030917

[20] Thrall PH, Burdon JJ (2000) Effect of resistance variation in a natural plant host-pathogen metapopulation on disease dynamics. Plant Pathology 49: 767–773.

[21] Laine A-L (2006) Evolution of host resistance: looking for coevolutionary hotspots at small spatial scales. Proceedings of the Royal Society B: Biological Sciences 273: 267–273. 16543168 10.1098/rspb.2005.3303 PMC1560038

[22] King KC, Delph LF, Jokela J, Lively CM (2009) The geographic mosaic of sex and the Red Queen. Current Biology 19: 1438–1441. 19631541 10.1016/j.cub.2009.06.062

[23] Berenos C, Wegner KM, Schmid-Hempel P (2011) Antagonistic coevolution with parasites maintains host genetic diversity: an experimental test. Proceedings of the Royal Society B: Biological Sciences 278: 218–224. 20685701 10.1098/rspb.2010.1211 PMC3013395

[24] Doligez A, Baril C, Joly HI (1998) Fine-scale spatial genetic structure with nonuniform distribution of individuals. Genetics 148: 905–920. 9504936 10.1093/genetics/148.2.905 PMC1459809

[25] Vekemans X, Hardy OJ (2004) New insights from fine-scale spatial genetic structure analyses in plant populations. Molecular Ecology 13: 921–935. 15012766 10.1046/j.1365-294x.2004.02076.x

[26] Howard RS, Lively CM (2002) The Ratchet and the Red Queen: the maintenance of sex in parasites. Journal of Evolutionary Biology 15: 648–656.

[27] Galvani AP, Coleman RM, Ferguson NM (2003) The maintenance of sex in parasites. Proceedings of the Royal Society of London Series B: Biological Sciences 270: 19–28. 12590767 10.1098/rspb.2002.2182 PMC1691212

[28] Roelfs AP, Groth JV (1980) A comparison of virulence phenotypes in wheat-stem rust populations reproducing sexually and asexually. Phytopathology 70: 855–862.

[29] Groth JV, McCain JW, Roelfs AP (1995) Virulence and isozyme diversity of sexual versus asexual collections of *Uromyces appendiculatus* (bean rust fungus). Heredity 75: 234–242.

[30] Gandon S, Nuismer SL (2009) Interactions between genetic drift, gene flow, and selection mosaics drive parasite local adaptation. American Naturalist 173: 212–224. 10.1086/593706 20374141

[31] Garant D, Forde SE, Hendry AP (2007) The multifarious effects of dispersal and gene flow on contemporary adaptation. Functional Ecology 21: 434–443.

[32] Parker MA (1991) Nonadaptive evolution and disease resistance in an annual legume. Evolution 45: 1209–1217. 28564187 10.1111/j.1558-5646.1991.tb04387.x

[33] Lande R, Arnold SJ (1983) The measurement of selection on correlated characters. Evolution 37: 1210–1226. 28556011 10.1111/j.1558-5646.1983.tb00236.x

[34] Thrall PH, Burdon JJ (1999) The spatial scale of pathogen dispersal: Consequences for disease dynamics and persistence. Evolutionary Ecology Research 1: 681–701.

[35] Lively CM, Delph LF, Dybdahl MF, Jokela J (2008) Experimental test for a co-evolutionary hotspot in a host-parasite interaction. Evolutionary Ecology Research 10: 95–103.

[36] Burdon JJ, Thrall PH, Brown AHD (1999) Resistance and virulence structure in two *Linum marginale*-*Melampsora lini* host-pathogen metapopulations with different mating systems. Evolution 53: 704–716. 28565630 10.1111/j.1558-5646.1999.tb05365.x

[37] Barrett LG, Thrall PH, Burdon JJ, Nicotra AB, Linde CC (2008) Population structure and diversity in sexual and asexual populations of the pathogenic fungus *Melampsora lini* . Molecular Ecology 17: 3401–3415. 18573166 10.1111/j.1365-294X.2008.03843.x PMC2653454

[38] Burdon JJ, Roberts JK (1995) The population genetic structure of the rust fungus *Melampsora lini* as revealed by pathogenicity, isozyme and RFLP markers. Plant Pathology 44: 270–278.

[39] Barrett LG, Thrall PH, Burdon JJ (2007) Evolutionary diversification through hybridization in a wild host-pathogen interaction. Evolution 61: 1613–1621. 17598744 10.1111/j.1558-5646.2007.00141.x

[40] Lawrence GJ, Burdon JJ (1989) Flax rust from *Linum marginale* - variation in a natural host pathogen interaction. Canadian Journal of Botany/Revue Canadienne de Botanique 67: 3192–3198.

[41] Jarosz AM, Burdon JJ (1991) Host-pathogen interactions in natural populations of *Linum marginale* and *Melampsora lini*. II. Local and regional variation in patterns of resistance and racial structure. Evolution 45: 1618–1627. 28564135 10.1111/j.1558-5646.1991.tb02667.x

[42] Burdon JJ, Jarosz AM (1991) Host-pathogen interactions in natural populations of *Linum marginale* and *Melampsora lini*. I. patterns of resistance and racial variation in a large host population. Evolution 45: 205–217. 28564067 10.1111/j.1558-5646.1991.tb05278.x

[43] Burdon JJ (1994) The distribution and origin of genes for race-specific resistance to *Melampsora lini* in *Linum marginale* . Evolution 48: 1564–1575. 28568407 10.1111/j.1558-5646.1994.tb02196.x

[44] Jarosz AM, Burdon JJ (1992) Host-pathogen interactions in natural populations of *Linum marginale* and *Melampsora lini*. III. Influence of pathogen epidemics on host survivorship and flower production. Oecologia 89: 53–61. 28313395 10.1007/BF00319015

[45] Ravensdale M, Nemri A, Thrall PH, Ellis JG, Dodds PN (2010) Co-evolutionary interactions between host resistance and pathogen effector genes in flax rust disease. Molecular Plant Pathology 12: 93–102. 21118351 10.1111/j.1364-3703.2010.00657.x PMC2999005

[46] Antonovics J, Thrall PH, Burdon JJ, Laine A-L (2011) Partial resistance in the *Linum-Melampsora* host-pathogen system: does partial resistance make the Red Queen run slower? Evolution 65: 512–522. 21029078 10.1111/j.1558-5646.2010.01146.x PMC3155823

[47] Cloutier S, Niu Z, Datla R, Duguid S (2009) Development and analysis of EST-SSRs for flax (*Linum usitatissimum* L.). Theoretical and Applied Genetics 119: 53–63. 19357828 10.1007/s00122-009-1016-3

[48] Peakall R, Smouse PE (2006) GENALEX 6: genetic analysis in Excel. Population genetic software for teaching and research. Molecular Ecology Notes. 288–295. 10.1093/bioinformatics/bts460 PMC3463245 22820204

[49] Littell RC, Milliken GA, Stroup WW, Wolfinger RD, Schabenberger O (2006) *SAS® for Mixed Models*. Cary, USA. : SAS Institute Inc.

[50] Lloyd M (1967) Mean crowding. Journal of Animal Ecology 36: 1–30.

[51] Degen B, Petit R, Kremer A (2001) SGS- Spatial Genetic Software: a computer program for analysis of spatial genetic and phenotypic structures of individuals and populations. Journal of Heredity 92: 447–448. 11773257 10.1093/jhered/92.5.447

[52] Deichsel G, Trampisch H (1985) Clusteranalyse und Diskriminanzanalyse. Gustav Fischer Verlag. Stuttgart.

[53] Thompson JN, Thompson JN (1994) The coevolutionary process. The coevolutionary process: xi+376p.

[54] Lawrence GJ, Dodds PN, Ellis JG (2007) Rust of flax and linseed caused by *Melampsora lini* . Molecular Plant Pathology 8: 349–364. 20507505 10.1111/j.1364-3703.2007.00405.x

[55] Kawecki TJ, Ebert D (2004) Conceptual issues in local adaptation. Ecology Letters 7: 1225–1241.

[56] Thrall PH, Burdon JJ, Bever JD (2002) Local adaptation in the *Linum marginale*-*Melampsora lini* host-pathogen interaction. Evolution 56: 1340–1351. 12206236 10.1111/j.0014-3820.2002.tb01448.x

[57] Flor HH (1942) Inheritance of pathogenicity in *Melampsora lini* . Phytopathology 32: 653–669.

[58] Clay K, Kover PX (1996) The Red Queen hypothesis and plant/pathogen interactions. Annual Review of Phytopathology 34: 29–50. 10.1146/annurev.phyto.34.1.29 15012533

[59] Ooi K, Yahara T (1999) Genetic variation of geminiviruses: comparison between sexual and asexual host plant populations. Molecular Ecology 8: 89–97.

[60] Thrall PH, Laine A-L, Ravensdale M, Nemri A, Dodds PN, et al (2012) Rapid genetic change underpins antagonistic coevolution in a natural host-pathogen metapopulation. Ecology Letters 15: 425–435. 22372578 10.1111/j.1461-0248.2012.01749.x PMC3319837

[61] Hoebe PN, Stift M, Holub EB, Mable BK (2011) The effect of mating system on growth of *Arabidopsis lyrata* in response to inoculation with the biotrophic parasite *Albugo candida* . Journal of Evolutionary Biology 24: 391–401. 21091813 10.1111/j.1420-9101.2010.02177.x

[62] Koslow JM, DeAngelis DL (2006) Host mating system and the prevalence of disease in a plant population. Proceedings of the Royal Society B: Biological Sciences 273: 1825–1831. 16790417 10.1098/rspb.2006.3519 PMC1634794

[63] Jokela J, Dybdahl MF, Lively CM (2009) The maintenance of sex, clonal dynamics, and host-parasite coevolution in a mixed population of sexual and asexual snails. American Naturalist 174: S43–S53. 10.1086/599080 19441961

[64] Neiman M, Koskella B (2009) Sex and the Red Queen. Lost Sex: Springer Netherlands. 133–159.

[65] Koskella B, Lively CM (2009) Evidence for negative frequency-dependent selection during experimental coevolution of a freshwater snail and a sterilizing trematode. Evolution 63: 2213–2221. 19473396 10.1111/j.1558-5646.2009.00711.x

[66] Lively CM (2010) A review of Red Queen models for the persistence of obligate sexual reproduction. Journal of Heredity 101: S13–20. 20421322 10.1093/jhered/esq010

[67] Dodds PN, Lawrence GJ, Ellis JG (2001) Contrasting modes of evolution acting on the complex *N* locus for rust resistance in flax. Plant Journal 27: 439–453. 11576428 10.1046/j.1365-313x.2001.01114.x

[68] Ellis JG, Lawrence GJ, Dodds PN (2007) Further analysis of gene-for-gene disease resistance specificity in flax. Molecular Plant Pathology 8: 103–109. 20507482 10.1111/j.1364-3703.2006.00375.x

[69] Lawrence GJ, Anderson PA, Dodds PN, Ellis JG (2010) Relationships between rust resistance genes at the *M* locus in flax. Molecular Plant Pathology 11: 19–32. 20078773 10.1111/j.1364-3703.2009.00563.x PMC6640504

[70] Hamilton WD (1980) Sex versus non-sex versus parasite. Oikos 35: 282–290.

[71] Bell G (1982) The masterpiece of nature: the evolution and genetics of sexuality. Berkeley, CA: University of California Press.

[72] Lively CM, Howard RS (1994) Selection by parasites for clonal diversity and mixed mating. Philosophical Transactions of the Royal Society B: Biological Sciences 346: 271–280. 10.1098/rstb.1994.0144 7708824

[73] Agrawal AF, Lively CM (2001) Parasites and the evolution of self-fertilization. Evolution 55: 869–879. 11430647 10.1554/0014-3820(2001)055[0869:pateos]2.0.co;2

[74] Steets JA, Wolf DE, Auld JR, Ashman T-L (2007) The role of natural enemies in the expression and evolution of mixed mating in hermaphroditic plants and animals. Evolution 61: 2043–2055. 17767581 10.1111/j.1558-5646.2007.00184.x

[75] Garcia-Guzman G, Morales E (2007) Life-history strategies of plant pathogens: distribution patterns and phylogenetic analysis. Ecology 88: 589–596. 17503586 10.1890/05-1174

