# Supplementary figures and images for: Population Processes at Multiple Spatial Scales Maintain Diversity and Adaptation in the Linum marginale - Melampsora lini Association
Source: PLoS One. 2012 Jul 31;7(7):e41366. doi: 10.1371/journal.pone.0041366 (PMC3409196; doi:10.1371/journal.pone.0041366)

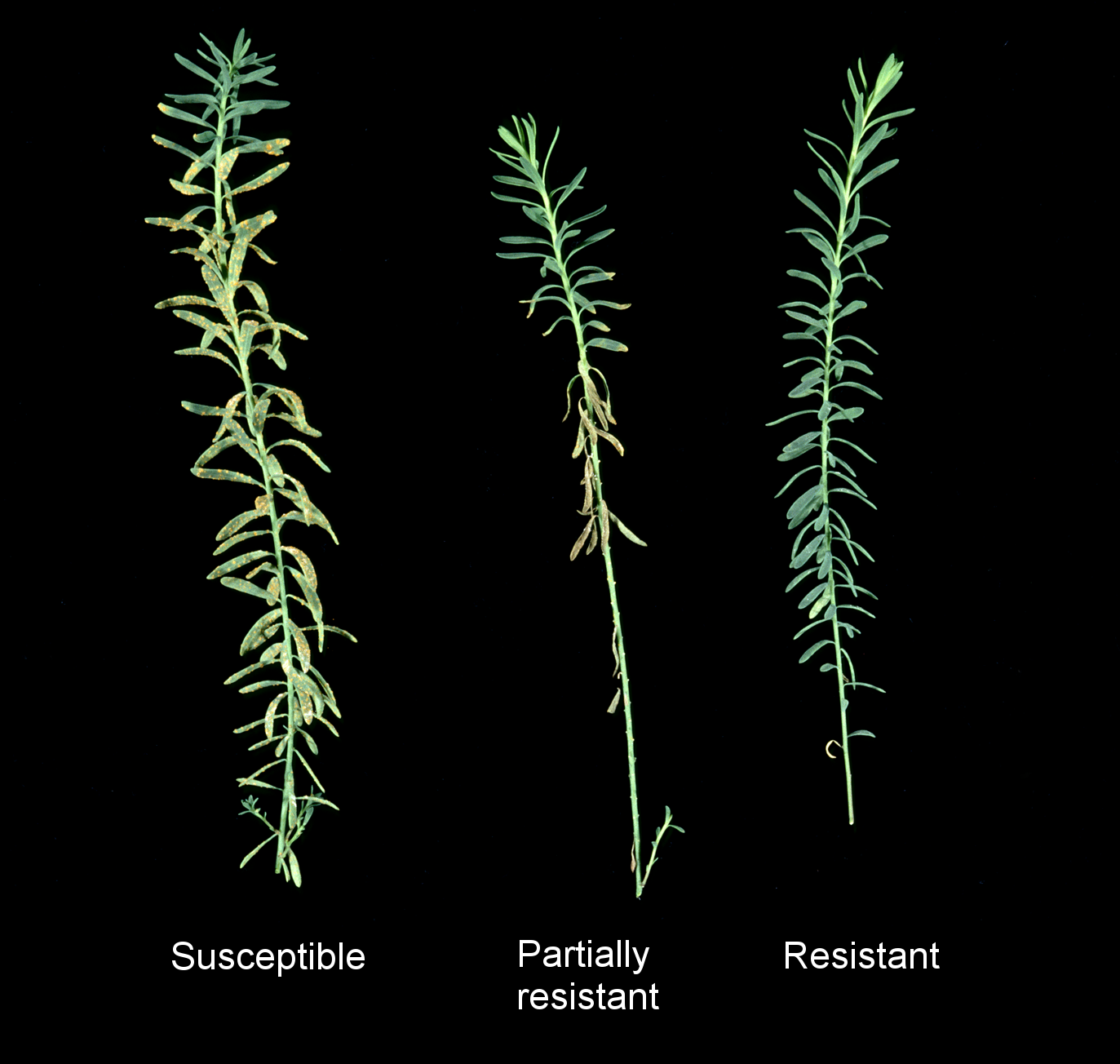

Supplement: Figure S1 — Differential responses to inoculation of wild flax ( Linum marginale ) genotypes with a flax rust ( Melampsora lini ) isolate. (TIF) [file pone.0041366.s001.tif]

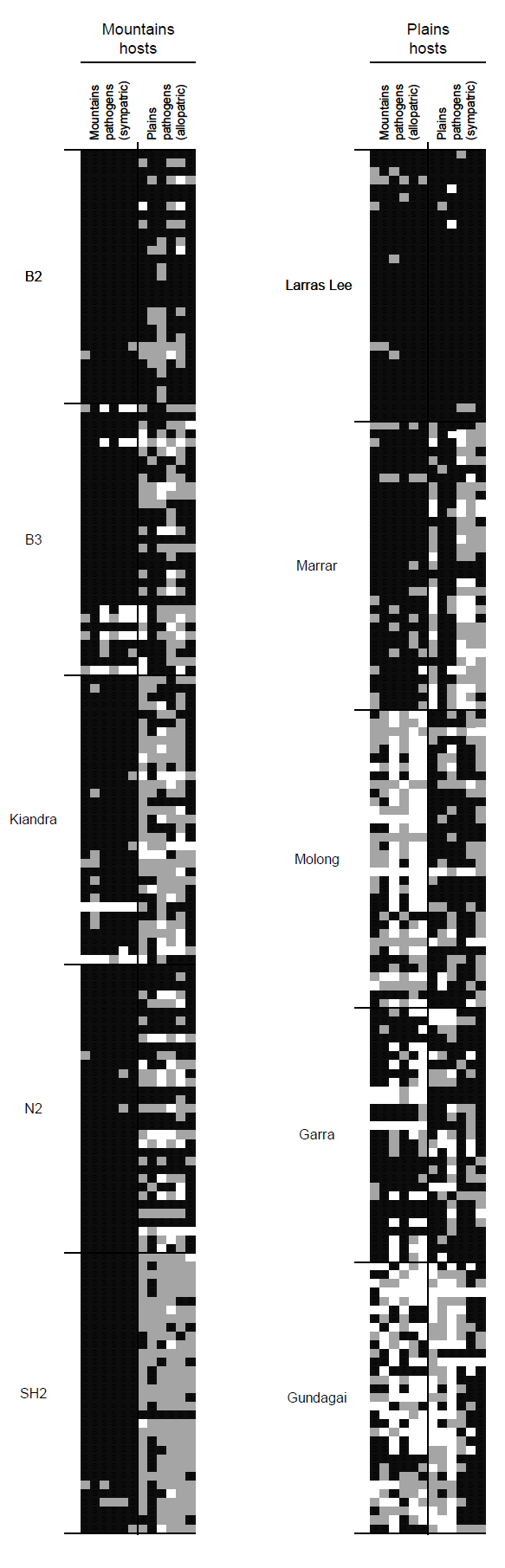

Supplement: Figure S2 — Regional adaptation of pathogen to their hosts. Linum marginale individual hosts from 5 Mountains (left panel) and 5 Plains populations (right panel) were inoculated with 6 Melampsora lini isolates from the Mountains (from left to right in columns: B3-4, B3-16, K8, N2-9, SH2-1, SH2-8) and 6 isolates from the Plains (from left to right in columns: L.Lee-19, Mar-34, Mar-41, Gar-26, Gun-14, Gun-37). Infections were scored as resistant (white squares), partially resistant (grey squares) and fully susceptible (black squares). (TIF) [file pone.0041366.s002.tif]

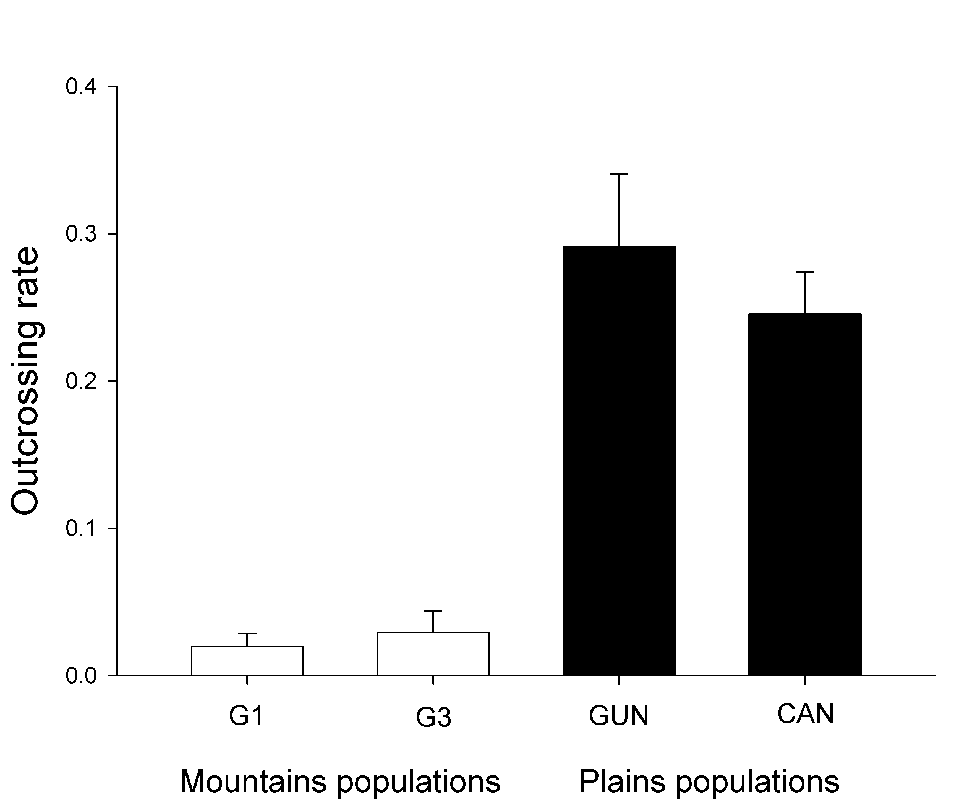

Supplement: Figure S4 — Estimates of outcrossing rates in two representative Mountains and Plains populations. Plots show the estimated population equilibrium outcrossing rates for Mountains populations G1 and G3 and Plains populations Gundagai and Canowindra calculated using 3–4 markers and the standard error of the mean. (TIF) [file pone.0041366.s004.tif]
