# Supplementary material for: Population Processes at Multiple Spatial Scales Maintain Diversity and Adaptation in the Linum marginale - Melampsora lini Association
Source: PLoS One. 2012 Jul 31;7(7):e41366. doi: 10.1371/journal.pone.0041366 (PMC3409196; doi:10.1371/journal.pone.0041366)
Supplement: Table S1 — Nucleotide sequences of microsatellites markers used in this study and size in bp of polymorphic alleles in the L. marginale populations assayed. (PDF) [file pone.0041366.s005.pdf]

Id	Locus	Primer pair	Number of polymorphic alleles	Sizes of polymorphic alleles (bp)
1	*Lub4*	5'-TGGAAGTCAACGAGATCGAA<br>5'-ACAGCAGCCTCCGTGTTTAT	2	252, 261
2	*Lub11*	5'-CCATGGGATGAAAATTCGAG<br>5'-CTTCACGAGGGGGAATGTTA	6	192, 195, 198, 199, 202, 204
3	*Lua58*	5'-CACCACCACCACAGTTTCTG<br>5'-AGGAACTCAGAGAGGCAGCA	5	201, 206, 209, 216, 219
4	*Lua64*	5'-GATCTCGCTCCCAACTACCA<br>5'-AGAGAAGGGGGACAAATTGC	6	206, 208, 211, 215, 220, 224
5	*Lua83B*	5'-CCCTCATTTTTCTCCTTCCA<br>5'-CAGGCGTTACAGTTTCCCATA	4	209, 213, 215, 227
6	*Lua105*	5'-CACCGTTAACTTCGCCATCT<br>5'-AAATGATGGATGGGATTGGA	1	229
7	*Lua133*	5'-ATCGCTCCTCTCTCCCTCTC<br>5'-GCTTTCTCAAGGGTGGAACA	8	222, 226, 228, 230, 231, 232, 233, 236
8	*Lu139*	5'-GCACGTAGGTGTTCTTATGCAA<br>5'-AGCTTCTTTCAGACGCCATC	2	272, 277
9	*Lu158*	5'-ATGCTTCTTCCACGTCTGCT<br>5'-GCTTGGACTCGAACAAGGAG	3	289, 291, 297
10	*Lu176*	5'-TCCATCCTCTGCATTTGTGA<br>5'-AAGACGAGTGCCCATTCCTA	4	546, 548, 549, 552
