# Supplementary material for: Population Processes at Multiple Spatial Scales Maintain Diversity and Adaptation in the Linum marginale - Melampsora lini Association
Source: PLoS One. 2012 Jul 31;7(7):e41366. doi: 10.1371/journal.pone.0041366 (PMC3409196; doi:10.1371/journal.pone.0041366)
Supplement: Table S2 — Plains and Mountains L. marginale populations used in this study with indices of genetic variability and polymorphic alleles for each population. (PDF) [file pone.0041366.s006.pdf]

Region	Regional sample size	Individual populations	Population sample size	A'	H'	Microsatellite loci with sizes of polymorphic alleles (bp)																																								
						Lub4		*Lub11*						*Lua58*					*Lua64*						*Lua83B*				*Lua105*	*Lua133*								*Lu139*		*Lu158*			*Lu176*			
						252	261	192	195	198	199	202	204	201	206	209	216	219	206	208	211	215	220	224	209	213	215	227	229	222	226	228	230	231	232	233	236	272	277	289	291	297	546	548	549	552
Mountains	668	B2	18	0.52	0.29	.	.	.	.	.	.	.	.			+	+	+	+	+	+	+	+			+	+		+		+	+	+	+	+		+									
		B3	29	0.52	0.33	.	.	.	.	.	.	.	.	+		+	+	+		+	+	+	+	+								+	+		+		+				+	+	+	+		
		K	24	0.58	0.28	.	.	.	.	.	.	.	.	+	+		+		+	+	+	+	+		+			+			+	+		+	+			+	+	+	+					+
		SH2	27	0.39	0.36	.	.	.	.	.	.	.	.	+		+	+			+	+	+					+		+				+		+						+		+	+		
		G1	297	0.76	0.36	+	+	+	+	+	+	+		+			+	+	+	+	+	+	+	+	+	+	+	+	+		+	+	+	+	+	+		+	+		+	+				
		G3	273	0.73	0.39	+	+			+	+	+	+	+	+		+	+	+	+	+	+	+	+	+	+	+		+		+	+	+	+	+	+		+	+	+	+					
Plains	630	Garra	31	0.73	0.39	.	.	.	.	.	.	.	.	+	+	+	+	+	+	+	+	+	+	+				+	+		+	+	+	+	+		+	+	+			+		+		+
		Marrar	30	0.67	0.46	.	.	.	.	.	.	.	.	+		+				+	+	+	+	+	+	+	+			+	+	+	+	+	+	+		+	+				+	+		+
		Molong	33	0.67	0.48	.	.	.	.	.	.	.	.	+	+		+	+		+	+	+	+	+	+			+		+	+	+	+	+	+				+				+	+	+	+
		Canowindra	229	0.73	0.40	+	+	+		+	+	+	+	+	+	+	+	+	+	+	+	+			+	+	+	+	+		+	+	+	+	+	+		+	+			+				
		Gundagai	307	0.68	0.47			+	+	+	+	+	+	+		+	+		+	+	+	+			+	+	+	+		+	+	+	+	+	+	+		+	+		+	+				

(+): allele is polymorphic in this population (scored as presence/absence); (.) non assessed

A': mean proportion of polymorphic alleles over total number of alleles scored per population; H': mean proportion of alleles present in each individual over total number of alleles scored averaged across all loci per population.
